# Computational drug design strategies applied to the modelling of human immunodeficiency virus-1 reverse transcriptase inhibitors

**DOI:** 10.1590/0074-02760150239

**Published:** 2015-11

**Authors:** Lucianna Helene Santos, Rafaela Salgado Ferreira, Ernesto Raúl Caffarena

**Affiliations:** 1 Fundação Oswaldo Cruz, Programa de Computação Científica, Rio de Janeiro, RJ, Brasil; 2 Universidade Federal de Minas Gerais, Instituto de Ciências Biológicas, Belo Horizonte, MG, Brasil

**Keywords:** HIV-1, computer-aided drug design, reverse transcriptase inhibitors, molecular modelling

## Abstract

Reverse transcriptase (RT) is a multifunctional enzyme in the human immunodeficiency virus (HIV)-1 life cycle and represents a primary target for drug discovery efforts against HIV-1 infection. Two classes of RT inhibitors, the nucleoside RT inhibitors (NRTIs) and the nonnucleoside transcriptase inhibitors are prominently used in the highly active antiretroviral therapy in combination with other anti-HIV drugs. However, the rapid emergence of drug-resistant viral strains has limited the successful rate of the anti-HIV agents. Computational methods are a significant part of the drug design process and indispensable to study drug resistance. In this review, recent advances in computer-aided drug design for the rational design of new compounds against HIV-1 RT using methods such as molecular docking, molecular dynamics, free energy calculations, quantitative structure-activity relationships, pharmacophore modelling and absorption, distribution, metabolism, excretion and toxicity prediction are discussed. Successful applications of these methodologies are also highlighted.

Established in 1983 as the causative agent of the acquired immune deficiency syndrome (AIDS) (Barre-Sinoussi et al. 1983), the human immunodeficiency virus (HIV) remains a worldwide health care issue. HIV has two known variants: HIV-1, which causes HIV infections worldwide, and HIV-2, mostly confined to West Africa (Reeves & Doms 2002). Thirty years of research and technological innovation have allowed validation of several steps of the HIV life cycle as intervention points for antiretroviral therapies. The highly active antiretroviral therapy (ART) is the standard treatment for HIV-infected patients and consists of the combination of three or more HIV drugs to reach maximal virological response and reduce the potential development of antiviral resistance (Asahchop et al. 2012). Currently, 26 antiretroviral drugs have been approved by the United States Food and Drug Administration (FDA) (FDA 2014).

Although the currently available ART proved that HIV infection is treatable, some challenges remain (Broder 2010). One important factor is the constant occurrence of new infections in many parts of the world. According to the Joint United Nations Programme on HIV/AIDS, approximately 35 million people were living with HIV and an estimated 2.3 million new HIV infections happened globally in 2012 (UNAIDS 2013). The life-long treatment brings another challenge. It can lead to long-term cardiac and metabolic complications such as dyslipidemias, insulin resistance, lipodystrophy, heart diseases and other related disorders (Filardi et al. 2008,Silverberg et al. 2009). Also, treatment can be impaired by the development of drug resistance strains when viral suppression is not maintained (Scarth et al. 2011). A vast number of viruses are produced daily in an infected individual and genetic variation within individuals has contributed to the emergence of diverse HIV-1 subtypes, complicating extensively the development of active drugs (Sarafianos et al. 2004). Therefore, current antiretroviral research efforts have been aiming at refining present therapies and discovering new drugs with lower toxicity and favourable resistance profile (Ghosh et al. 2008,^2011^, Maga et al. 2010, Quashie et al. 2012,Cao et al. 2014, Michailidis et al. 2014).

Presently, computational methods are an important part of the drug design process and this kind of modelling is often denoted as computer-aided drug design (CADD). Computational methods can offer detailed information about the interaction between compounds and targets, increasing the efficiency and lowering the cost of research in several stages of drug discovery (Kirchmair et al. 2011). Choosing the most appropriate computational technique to apply when planning novel drugs depends on the understanding of the target of interest (Jorgensen 2004). So far, various computational methods have been employed to the development of anti-viral drugs [reviewed by Kirchmair et al. (2011) and Wlodawer (2002)]. It is noteworthy that some approved drugs for the treatment of an assortment of diseases owe their discovery in part to CADD methods [recently reviewed by Sliwoski et al. (2014)]. This group includes anti-HIV drugs such as protease inhibitors saquinavir (Invirase^®^), ritonavir (Norvir^®^) and indinavir (Crixivan^®^), integrase inhibitor raltegravir (Isentress^®^), reverse transcriptase (RT) inhibitor rilpivirine (RPV) (Edurant^®^) and fusion inhibitor enfuvirtide (Fuzeon^®^).

The goal of the present review is to give an overview of CADD methods, the challenges involved and current innovations when modelling one of the HIV-1 enzymes: the RT.

## HIV-1 RT enzyme and inhibitors

**Fig. 1:**
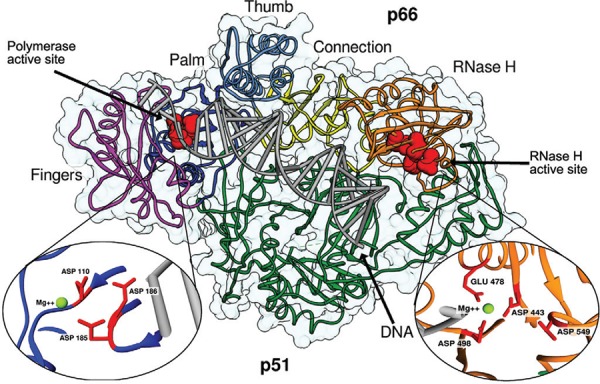
structure of human immunodeficiency virus-1 reverse transcriptase in complex with DNA [Protein Data Bank code: 1T05 (Tuske et al. 2004)]. The two domains are the p66 (coloured) and the p51 (green). The polymerase domain displays a highly conserved structure that resembles the shape of the human right hand, consisting of fingers domain (magenta), palm domain (blue), thumb domain (light blue). The p66 subunit also includes the connection domain (yellow) and ribonuclease H (RNase H) domain (orange). The polymerase active site is located in the canter of palm, fingers and thumb subdomains. The three catalytic aspartic acid residues (110, 185 and 186), shown in red, are located in the palm subdomain and bind the cofactor divalent ion (Mg2+). The RNase H domain is situated at the p66 C-terminus, approximately 60 Å from polymerase active site. The RNase H active site contains a DDE motif comprising the carboxylates residues ASP443, GLU478, ASP498 and ASP549 that can coordinate a divalent Mg2+ ion.

The HIV-1 enzyme RT is a primary target for antiretroviral drugs. Today, 13 inhibitors act against it, including the very first drug used in HIV treatment, the nucleoside RT inhibitor (NRTI) zidovudine (AZT) (Retrovir^®^) (Esposito et al. 2012). RT is the enzyme that converts the single-stranded RNA viral genome into a double-stranded DNA (dsDNA) provirus, which is afterwards imported into the cell nucleus to be integrated into the host chromosome with the help of integrase (Esposito et al. 2012), another HIV enzyme. Other crucial activities of the retrotranscription process can be attributed to this highly dynamic enzyme: an endonucleolytic ribonuclease H (RNase H) activity and strand transfer (Liu et al. 2008). RT is a heterodimer (Fig. 1) composed of two subunits of 560 and 440 amino acid (aa) residues, referred to as p66 and p51, respectively (Menendez-Arias 2013). These subunits share almost the same aa sequences. However, p51 lacks the catalytic activity and the RNase H domain, performing a structural role (Kohlstaedt et al. 1992). Unlike p51, p66 has a more flexible structure and contains the polymerase and RNase H active sites ( Kohlstaedt et al. 1992). Although, all the commercially available RT-targeting drugs affect the polymerase activity inhibiting its function, some RNase H inhibitors have recently been designed and studied (Tramontano & Di Santo 2010, Distinto et al. 2013) (Steitz 1999, Tuske et al. 2004).

**Fig. 2A:**
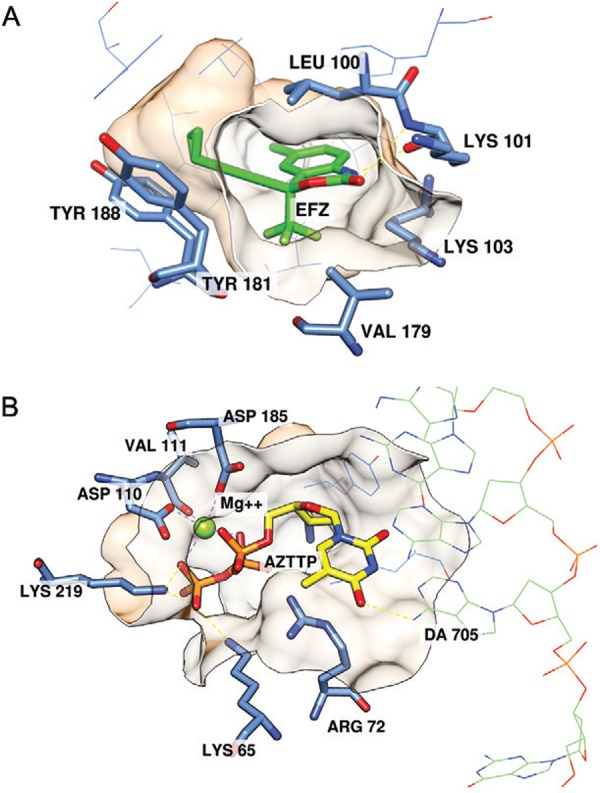
efavirenz (EFZ) (green) within the nonnucleoside reverse transcriptase inhibitor (NRTI) allosteric binding site [Protein Data Bank (PDB) code: 1FK9 (Ren et al. 2000b)]: B: zidovudine (AZT) (yellow) within the NRTI binding site [PDB code: 3V4I (Das et al. 2012)].

**Fig. 3:**
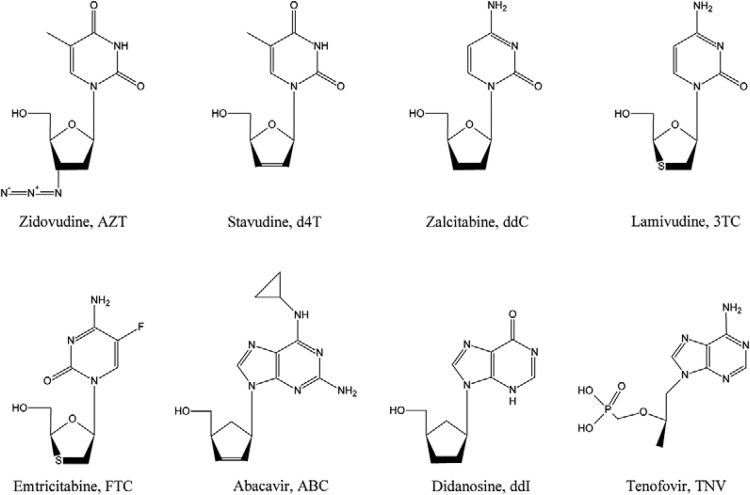
chemical structures of eight approved nucleoside and nucleotide reverse transcriptase inhibitors.

The two main classes of RTIs include NRTIs and nonnucleoside transcriptase inhibitors RTIs (NNRTIs). The NRTIs are composed of modified nucleosides that mimic and compete with natural substrates for binding and incorporation at the polymerase site (Fig. 2B) (De Clercq 2010). They act as chain terminators due to the lack of a 3'-OH group on their sugar moiety. Similarly to their natural counterparts, the NRTIs need to be converted in 5'-triphosphate nucleotides by host-cell kinases to compete with the analogous deoxynucleotide-triphosphates and consequently be incorporated into the growing DNA strand (Esposito et al. 2012). The current clinically available NRTIs are structurally similar to pyrimidine and purine analogues, including thymidine analogues AZT and stavudine (Zerit^®^); together with cytidine analogues zalcitabine (Hivid^®^), lamivudine (Epivir^®^) and emtricitabine (Emtriva^®^). Purine analogues include the inosine analogue didanosine (Videx^®^) along with the carbocyclic nucleoside analogue abacavir (Ziagen^®^), a guanine analogue when in its active form (Fig. 3) (Mehellou & De Clercq 2010).

In the NRTI class, there are RTIs that already have a phosphate group incorporated into their structure. Also known as nucleotide RTIs, such as tenofovir (TFV) (Fig. 3), formulated as TFV disoproxil fumarate (TDF) (Viread^®^), they require only two phosphorylation steps to achieve their active triphosphate derivatives (Squires 2001). However, their mode of action is the same as for the NRTIs.

**Fig. 4:**
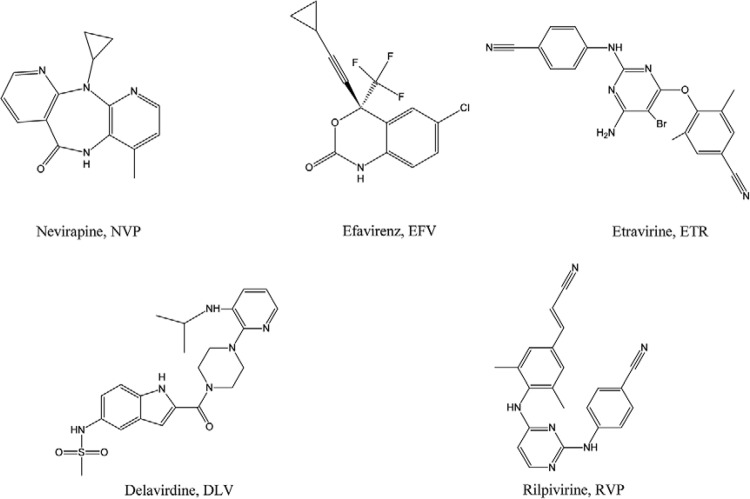
chemical structures of five approved nonnucleoside reverse transcriptase inhibitors.

The NNRTIs are allosteric inhibitors of DNA polymerisation. These compounds bind in a noncompetitive manner to a hydrophobic pocket (Fig. 2A) located approximately 10 Å away from the polymerase active site, causing conformation changes that impair DNA synthesis (Squires 2001). During the DNA synthesis, the RT fits a “closed” conformation bringing the fingers and thumb subdomains closer to the palm one and allowing the binding of nucleic acids. The presence of an NNRTI leads to an open conformation that restricts the thumb to a hyperextension position, which prevents the polymerisation (de Bethune 2010,Das et al. 2012). The currently approved NNRTIs are nevirapine (NVP) (Viramune^®^), efavirenz (Sustiva^®^), delavirdine (DLV) (Rescriptor^®^), etravirine (ETR) (Intelence^®^) and RPV (Fig. 4).

Despite their popularity and the number of drugs already approved for this class, most RTIs have their antiviral potency limited by several factors such as mutations in the binding site, drug-drug harmful interactions, toxicity and long-term complications (Ho & Hitchcock 1989, Waters et al. 2007, Johnson et al. 2008, Cihlar & Ray 2010). Consequently, new inhibitors are being sought out and, supported by the available knowledge of the RT structure and its known inhibitors, the field of drug design has been adequately applied to study and optimise lead compounds. RT has been the focus of extensive research, including several structural biology studies that resulted in the determination of numerous crystallographic structures. Currently, over 100 RT crystal structures are available in the Research Collaboratory for Structural Bioinformatics Protein Data Bank (PDB) repository (Berman et al. 2000). The available RT crystal structures provide insights into the conformational flexibility of the protein, including the conformational changes induced by inhibitor and DNA binding (Titmuss et al. 1999). For instance, the formation of the nonnucleoside inhibitor-binding pocket (NNIBP) is induced by the presence of an NNRTI, i.e., it only exists in RT structures complexed with this kind of inhibitors. The “open” and “closed” conformations can be found in crystal structures with bound and unbound DNA, respectively. The RT structures are alike, presenting some structural changes mainly in the binding pockets. Commonly, when combined with computational methods, crystallographic structures provide molecular insights into drug-target interactions and the mechanisms that set different drug responses. Computational studies, frequently applied in CADD, such as molecular docking, molecular dynamics (MD), free energy calculations, quantitative structure-activity relationships (QSARs), pharmacophore modelling and absorption, distribution, metabolism, excretion and toxicity (ADMET) have been performed using the RT and its inhibitors as targets. A successful example of the multidisciplinary effort in drug discovery, when modelling RTIs, is the 2011 FDA-approved NNRTI RPV. RPV was developed by combining chemical synthesis with broad antiviral screening; bioavailability and safety assessments in animals and molecular modelling, including analysis of three-dimensional (3D) structures and ligand-target relationships by molecular docking (Janssen et al. 2005).

## Molecular docking

A molecular docking study can provide a better understanding of the interactions between a protein and a ligand. Such applications of this method in finding lead compounds are described in details by Shoichet et al. (2002), Kroemer (2007) andCavasotto and Orry (2007). Docking begins with sampling ligands orientations and conformations within the target binding site [for reviews see Taylor et al. (2002), Moitessier et al. (2008), Meng et al. (2011) and Yuriev and Ramsland (2013)]. Afterwards, the best poses for each ligand are determined and the compounds are ranked according to a scoring function (Lahti et al. 2012). One of the earliest docking methods was constructed based on the lock-and-key theory of ligand-protein binding, where both the protein and ligand structures are treated as rigid bodies (Kuntz et al. 1982). Currently, the most popular docking programs address the ligand flexibility when binding to rigid targets, such as AutoDock (Goodsell et al. 1996), DOCK (Ewing et al. 2001), FlexX (Kramer et al. 1999), Glide (Friesner et al. 2004, Halgren et al. 2004), GOLD (Verdonk et al. 2003), Molegro Virtual Docker (Thomsen & Christensen 2006), AutoDock Vina (Trott & Olson 2010) and Surflex (Jain 2003, 2007, 2007, Spitzer & Jain 2012), to name a few.

The most explored RTIs are the NNRTIs, with a large number of chemical and structurally diverse compounds identified as genuine inhibitors that suppress HIV-1 replication (De Clercq 2009, de Bethune 2010). Although diverse, all compounds bind in the NNRTI binding pocket in similar conformation and manner (Zhan et al. 2013). The NNRTI binding pocket consists of hydrophobic residues with significant aromatic character (Y181, Y188, F227, W229, Y232 and Y318 of p66) and hydrophilic residues (K101, K103, S105, D192, E224 and H235 of p66 and E138 of p51) (Sluis-Cremer et al. 2004). The solvent accessible entrance is formed by the residues L100, K101, K103, V179, Y181 and E138 (Fig. 2A). However, this open state of the binding pocket is only noticeable when the structure is co-crystallised with NNRTIs, mainly due to significant torsional shifts of the Y181 and Y188 residues to accommodate the ligand (Hsiou et al. 1996). In the absence of a ligand, the binding pocket is blocked since the side chains of Y181 and Y188 are situated at the hydrophobic core, representing a closed state of the pocket. This inherent flexibility of the binding pocket provides a challenge to molecular docking. Previous docking studies showed that the difference in geometries can affect the accuracy of ligand binding energies when docking other NNRTIs into the inhibitor binding pocket (Smith et al. 1995, Titmuss et al. 1999). Numerous studies (Titmuss et al. 1999, Zhou et al. 2002, Ragno et al. 2005, Sherman et al. 2006, Ivetac & McCammon 2011) have reported the employment of molecular docking, by itself or in combination with other molecular modelling techniques, upon targeting the binding pocket with different approaches to ligand and receptor flexibility.

The search for novel anti-HIV inhibitors is also extended to natural products [reviewed by Asres et al. (2005) and Vo and Kim (2010)]. Historically, natural products have been a prolific source for lead drugs and continue to provide structural templates for drug discovery, since the majority of all market drugs have their origin *in nature* (Chin et al. 2006, Newman & Cragg 2012). However, only a few of anti-HIV natural products that have been reported to exhibit inhibition activities have reached clinical trial and so far none of them is commercially available (Asres et al. 2005). Recently, computer-aided approaches have found room in natural product research (Rollinger et al. 2006a, b, 2008) and some studies had RT as their target (Sangma et al. 2005, Ehrman et al. 2007, Seal et al. 2011, Ashok et al. 2015).

In an early work (Currens et al. 1996), a natural product extracted from the tropical rainforest tree *Calophyllum lanigerum*, calanolide A, showed promising results as an NNRTI. However, this natural product is difficult to purify from its natural source in a sufficient amount for clinical use and its low therapeutic index contributed to the delay of its clinical development. Lu et al. (2012)investigated a calanolide A analogue, 10-chloromethyl-11-demethyl-12-oxo-calanolide A (F18), using experimental and docking studies. F18 was chosen since it showed high potency against wild-type (WT) HIV-1 [half maximal effective concentration (EC_50_) = 7.4 nM] in a TZM-bl cell based assay. Docking studies were conducted using AutoDock 4.2 and the structures of F18 and NVP (control) were docked into three different RT crystal structures: WT [PDB code: 1VRT (Ren et al. 1995)], L100I mutant [PDB code: 1S1U (Ren et al. 2004)] and Y181C mutant [PDB code: 1JLB (Ren et al. 2001)]. The results showed that F18 had a rigid structure and restricted binding when compared to NVP. In the WT structure, no meaningful interactions between F18 and the binding pocket residues were predicted. On the other hand, an aromatic interaction between Y188 and NVP was observed, indicating that the WT structure is more sensitive to NVP than to F18. With the L100I structure, the binding pocket was altered and there was less hydrophobic interaction with F18 and thus, L100I mutation conferred moderate resistance to F18. However, Y181C structure was favourable to F18, since the change of tyrosine to cysteine permitted more spatial flexibility to the compound and increased antiviral activity. The docking analysis was later correlated with cell-based assays and both results indicated that F18 might bind to a distinct motif on the RT from that of NVP, which can be the cause of its drug resistance profile.

A molecular docking study by Allen et al. (2015) evaluated the latest version of the program DOCK with the SB2012 test set [expanded from the SB2010 (Mukherjee et al. 2010)] composed of a diverse range of receptors, including 21 RT-NNRTI crystal structures. All receptors were structurally aligned to facilitate docking all the ligands into all of them. Therefore, docking statistics was based on the crystallographic ligand and its pose prediction the root-mean-square deviation (RMSD) when docked into its native receptor (redocking) or a nonnative structure from the same drug-target family (cross-docking). Also, if any particular pose comparison achieved either an RMSD over 2.0 Å or a positive score, it was considered a nonviable reference and the pairing was not included in the docking statistics. Overall, the success rates for RT structures were 71.4%, whereas the success rate of redocking by itself was 95.2%. Scoring and sampling failure rates were 18.3% and 10.4%, respectively. However, there was a high incidence of nonviable pairings, 220 nonviable out of 441 pairings, which could be related to the conformation of the binding site and the presence six mutations (L100I, K101E, K103, E138K, Y181C and Y188C) known to confer resistance to NNRTIs in the set of structures. The strategy of starting the docking process from 3D structures of RT-NNRTI complexes is a very delicate one, not only due to the intrinsic flexibility of the RT, but also due to the allosteric binding pocket conformational changes to accommodate the NNRTIs (Tronchet & Seman 2003). In general, proteins go through conformational changes when performing their functions and the molecular recognition between a protein and small molecules involves structural flexibility (Ivetac & McCammon 2011). Recently, more advanced methods have introduced protein flexibility and its influence on ligand recognition, supported by the exponential growth in computer processing and disk capacity (Carlson & McCammon 2000, Cavasotto & Singh 2008).

An attempt to account for a small amount of plasticity of the receptor is to use soft scoring functions, capable of tolerating some overlapping between the ligand and the protein, but still maintaining a rigid receptor (Jiang & Kim 1991, Claussen et al. 2001). This implementation, which is known as soft docking, is computationally efficient since only the scoring parameters need to be changed whereas everything else remains unaltered when compared to rigid docking (B-Rao et al. 2009). Although, this option has been pursued due to its computational simplicity, it can introduce false-positives if the tolerance is set too high (Ivetac & McCammon 2011). Other strategies to incorporate receptor flexibility involve sampling of side-chain conformers within the binding pocket, with the use of a library of rotamers and the use of an ensemble of receptor structures (Cavasotto & Orry 2007).

Glide is one of the programs that uses soft docking receptors by scaling the van der Waals radii (Elokely & Doerksen 2013). In their study, Bahare and Ganguly (2014)evaluated the accuracy of their docking procedure consisting of the docking of the NNRTI TNK 651, extracted from a X-ray crystallographic RT structure [PDB code: 1RT2 (Hopkins et al. 1996)], by means of two different programs: Glide and FlexX. The first employs a hybrid approach that combines one or more docking algorithms in the generation of the ligand poses (Moitessier et al. 2008). The second is based on incremental construction, where the ligand is built dynamically in the active site, frequently counting on libraries of favoured conformations (Moitessier et al. 2008). The RMSD values between the docking prediction and the experimental conformation of TNK 651 were 0.370 Å and 1.254 Å, with Glide and FlexX, respectively.

Recently, Fraczek et al. (2013) assessed the ability of docking programs and their scoring functions to predict the relative biological activity of triazole NNRTIs. In total 111 known 1,2,4-triazole and 76 other azole type NNRTI were submitted to different docking protocols that involved softened van der Waals potentials (FlexX, Molegro Virtual Docker and Glide XP and SP), ligand flexibility (AutoDock Vina) and receptor flexibility employing the Induced Fit Docking (IFD) method (Sherman et al. 2006). The IFD method combines an iterative procedure to obtain initial poses allowing flexibility into rigid receptors, followed by a technique for modelling receptor conformational changes, present in the refinement module of Prime (Jacobson et al. 2002, 2004) program that explores flexibility. However, while the method allows efficient small backbone movements, it is inappropriate to more severe conformational changes due to an increase in complexity and computational cost (Ivetac & McCammon 2011). The RT structures 2RKI (Kirschberg et al. 2008), in complex with a triazole and 3DLG (Ren et al. 2008), in complex with a benzophenone, were used in the docking procedures. Since the core structure of the compounds is similar, the triazole binding mode was assumed as the reference pose. For 2RKI, all programs showed good predictions of ligand orientation in the binding site when compared to the reference pose, Glide SP (97.3%), Glide XP (59.5%), IFD (97.3%), AutoDock Vina (94.6%), FlexX (65.8%) and Molegro Virtual Docker (61.3%). The predictions for 3DLG were lower to most of them, Glide SP (82.9%), Glide XP (67.6%), IFD (88.3%), AutoDock Vina (75.7%), FlexX (36%) and Molegro Virtual Docker (71.2%). However, none of the scoring functions reached a perfect ranking of the compounds according to their activities. Glide XP achieved the highest correlation, Spearman's ρ of 0.7, which corresponds to around 0.75 probability of identifying the most active compound from two compounds. The outcomes from this study suggested that different docking methods can provide good binding mode predictions, yet results should not rely only on docking scores when trying to rank active compounds with different potencies.

Another approach considers a discrete number of receptor conformations (obtained either experimentally or by computational means) to represent the flexibility instead of making the protein flexible throughout the docking process (Knegtel et al. 1997, Osterberg et al. 2002, Huang & Zou 2007). This procedure is known as ensemble docking. Structure ensembles can diverge in their sidechain, loops and domain orientations. A study byMeleddu et al. (2014) performed ensemble docking experiments in an attempt to predict the binding mode of a compound from a series of dual inhibitors, a single molecule that is able to inhibit two enzymes activities, of RT-associated functions. Since the most promising compound showed activity in vitro against both the RNA-dependent DNA polymerase (RDDP) and RNase H of RT [half maximal inhibitory concentration (IC_50_) of 6 ± 2 μM and 4 ± 1 μM, respectively], docking was performed into six NNRTI bound structures [PDB codes: 1VRT, 2ZD1 (Das et al. 2008), 1EP4 (Ren et al. 2000c), 3QO9 (Das et al. 2011), 1RTI (Ren et al. 1995) and 1TV6 (Pata et al. 2004)] and one RNase H inhibitor bound structure [PDB codes: 3LP2 (Su et al. 2010)], using the QM-Polarized Ligand docking protocol. The 1TV6 structure was also considered for RNase H docking experiments, using the whole domain. Post-docking procedures based on energy minimisation and binding free energy [molecular mechanics with generalised Born and surface area solvation (MM-GBSA) calculations] were also performed. The best ensemble score was obtained in the WT structure 1RTI (GScore of -11.04 kcal/mol), however the best free energy of binding (-48.0 kcal/mol) when comparing MM-GBSA values were obtained in the mutated Y181C NNIBP of the 1TV6 structure. Poses in the RNase H binding sites achieved worse ensemble scores (> -7.50 kcal/mol) and free energy of binding than those in the NNRTI binding pocket (> -38.60 kcal/mol). Biochemical and modelling studies combined suggested that polymerase inhibition was due to the compound binding into the NNRTI pocket, where the RDDP activity was retained in all RT strains. Whereas, binding into an allosteric site close to RNase H catalytic residues might be responsible for RNase H inhibitory activity, since a single-point mutation inserted in this site decreased the inhibition of the RNase function by the compound. Therefore, the compound might behave as a dual-site dual-function inhibitor.

As significant as docking methods are in drug discovery, the search for potential drug candidates often initially requires screening libraries of available compounds to identify novel hits. This computational approach, referred to as virtual screening (VS) is an important drug discovery tool, which allows identification of lead compounds among large databases, thanks to its ability to discriminate between true and false-positives (Cummings et al. 2005). Several VS approaches have been described, among which the most common one uses molecular docking as a faster and more cost-effective alternative than experimental high-throughput screening. VS aims to reduce a vast virtual library of approximately 10^5^-10^6^ chemical compounds, to a more manageable number for experimental screening against biological targets and further synthesis of analogues, which could lead to potential drug candidates.

Herschhorn and Hizi (2008) conducted a VS study to identify novel NNRTIs from a commercially available library of 46,000 compounds (Tripos Leadquest3) against two RT crystal structures [PDB codes: 1FK9 (Ren et al. 2000b) and 1DTQ (Ren et al. 2000a)]. The library of “druglike” (Lipinski et al. 2001) compounds was docked into the two structures in parallel using Surflex, in a way that the difference in the results for both structures could be accounted. The molecules were ranked by their score according to the average of their top ten conformations. Compounds with exceptionally high scores or high ratio of docking score to the number of rotational bonds were all included in the list of potential compounds. Overall, 740 out of the 46,000 were selected, purchased and submitted to a primarily experimental test for inhibiting in vitro RDDP of recombinant HIV-1 RT. Only 71 of the selected compounds inhibited more than 84% of RT-associated RDDP at the tested concentration (50 μg/mL). A total of 17 novel compounds were later chosen to further experimental evaluations according to their high RT inhibition at nanomolar concentrations and structural diversity. Some of the molecules shared similar elements with the phenylethylthiazolylthiourea (PETT) series (Ahgren et al. 1995, Ren et al. 2000a), which are known NNRTIs, however instead of the original PETT pyridine rings, other chemical structures such as phenyl, furan or cyclohexane rings were found. The original inhibitor in the selected structure 1DTQ is a PETT derivative, showing that the docking process could retrieve compounds that resemble the native inhibitor found in the crystallographic structure.

An interesting report displays an example in which a new class of inhibitors was identified from VS; despite the fact that the compounds initially evaluated were false-positives. After reporting failure to yield active NNRTIs from their top-scoring compounds, Barreiro et al. (2007b) still pursued one of the scaffolds. VS was performed in a library of 70,000 compounds (Maybridge Library) using first a chemical similarity search, considering known NNRTIs as reference structures and then the subsequent library (2,000 molecules) was docked into a single RT structure [PDB code: 1RT4 (Ren et al. 1998)] using Glide 3.5. The top 100 scored compounds were later submitted to MD simulations to estimate the free energy of binding by means of the MM-GBSA method (Kollman et al. 2000), as well as to evaluate the change in free energy of hydration using the GBSA (Still et al. 1990). Finally, four top-scoring compounds were subjected to experimental evaluations and showed no ability to inhibit HIV replication. Nevertheless, the top-scoring compound, a confirmed false-positive of the VS procedure, was assessed by computational analysis and modifications were made in its structure, removing or adding functional groups to create analogues. These last sets of compounds provided more favourable results than the original one and some of them were reported to be potent anti-HIV agents (lowest IC_50_ = 0.31 μM). This study demonstrates the importance of chemical insights and that even compounds that do not inhibit an enzyme with detectable activity may provide a scaffold to find new inhibitors.

In a recent study, Chander et al. (2015)performed a VS study to identify novel NNRTIs that could potentially act against WT and drug resistance RT strains. Firstly, a screening of 30,000 molecules, extracted from the Maybridge Library and filtered by the Lipinski Rule of Five (Lipinski et al. 2001), were performed by the Glide high-throughput VS module against a WT RT structure [PDB code: 4G1Q (Kuroda et al. 2013)]. Afterwards, compounds in the top 10% were retrieved and submitted to docking into the WT structure using the Glide SP module. This procedure was once again performed with the Glide XP module. After all, the top 30 scored hits were subjected to another round of docking into the RT mutant strains K103N [PDB code: 3TAM (Gomez et al. 2011)] and K103N/Y181C [PDB code: 4I2Q (Johnson et al. 2012)]. Out of 30 compounds, around nine exhibited good binding modes and hydrophobic interaction with binding site residues Y181, Y188, F227, W229 and Y318 toward all three RT strains. Hydrogen bonding interaction with the residue K101 was also presented for the majority of the hit compounds. Although no experimental studies were conducted, all nine compounds had favourable predictions for ADMET properties.

In the next section, we discuss works where MD simulations alone or in combination with other methods were applied to RT systems.

## MD

MD is a powerful and extensively used method to gather information on the dynamical properties and processes of proteins and other biological macromolecules, also time-dependent and thermodynamical information (Adcock & McCammon 2006). It is a commonly employed tool in a vast number of fields such as structural biochemistry, biophysics, molecular biology and pharmaceutical industry (Galeazzi 2009). MD simulations have a broad range of usage. For instance, they are extensively employed to refine experimental or model-derived protein structures, to inspect the strength and stability protein-ligand complexes resulting from a docking study, to aid drug discovery and many more (Lahti et al. 2012).

In MD simulations, physical movements of atoms and molecules are portrayed over time, usually over tens to hundreds of nanoseconds (ns) reaching up to milliseconds, a feat provided by iterative calculations of the forces present that act on the system (a complex of protein, ligand, solvent and often a lipid bilayer) and the consequential movements (Adcock & McCammon 2006). A successful MD simulation depends on the choice of a suitable energy function for describing the inter and intramolecular interactions (Galeazzi 2009). Forces between atoms and the potential energy of the system are described by the force fields, well-parameterised functions obtained from experimental or quantum mechanical studies. Widely applied force fields included several versions from OPLS-AA (Jorgensen et al. 1996), CHARMM (MacKerell et al. 1998), AMBER (Cornell et al. 1995) and GROMOS (Oostenbrink et al. 2004). Common MD softwares are GROMACS (Van Der Spoel et al. 2005), AMBER (Pearlman et al. 1995) and NAMD (Kale et al. 1999).

RT flexibility is essential for the polymerisation and RNase H activities, in addition to inhibition of enzymatic activity. Madrid et al. (2001) conducted a study to analyse flexibility for two RT systems, bound [PDB code: 2HMI (Ding et al. 1998)] and unbound [PDB code: 1DLO (Hsiou et al. 1996)] to dsDNA, by means of MD simulations. MD simulations of 125 ps, with an integration step of 1 fs, were performed using AMBER keeping DNA and protein unrestrained in solution. From the simulations analysis, it was concluded that the RT flexibility depends on its ligation state. The complex RT/dsDNA showed more flexible regions than the unbound RT, particularly in the fingers and thumb p66 subdomains. This outcome was consistent with the conformation changes found in crystallographic structures and biochemical data. Although the simulation times to the systems were very short, probably due to hardware limitations at that time, these simulations showed that it is possible to complement the RT information available from existing crystal structures by means of MD.

A few years later, Ivetac and McCammon (2009)published an impressive paper focused on the inhibition mechanism of NNRTIs, using structures from crystallographic and MD data, through multicopy MD simulations (cumulative total simulation time of 360 ns). Principal components analysis (PCA) were employed to interpret the dynamics from both a crystallographic ensemble of 13 RT structures (1 apo, 2 substrate-bound and 10 NNRTI-bound) and a MD ensemble from three simulated systems (RT with NNRTI binding pocket closed, open and bound to NVP). PCA has been performed previously on other proteins for which substantial crystallographic data exists (van Aalten et al. 1997, Gorfe et al. 2008). Comparison of the systems showed similar movements, characterised by opening/closing of the fingers and thumb subdomains, between NNRTI-free simulations and crystallographic ensemble and quite distinct of those of the NNRTI bound simulations. The fingers and thumb subdomains in the NNRTI bound simulation made movements roughly orthogonal to those presented by the other simulated systems. This difference might demonstrate that the effect of an NNRTI is to constrain the motion between these subdomains. Consequently, NNRTIs may act as “molecular wedges” sterically blocking the full range of the subdomain movements since the NNRTI binding pocket is located proximally of their hinge points. The time scale of the simulations and the multicopy approach, chosen in this work, helped enhance the sampling of the dominant motions found in the ensembles.

Both studies displayed the use of MD simulations to understand RT flexibility. However, MD simulations and docking are usually employed as complementarily methods. While docking techniques allows for a vast exploration of ligand conformation and screening of large libraries in a short time, MD simulations can be employed to optimise conformations of the receptor-ligand complex, explore other receptor conformations and achieve accurate binding free energy predictions (Alonso et al. 2006).

Methods to consider receptor flexibility are also employed in VS, commonly using conformation ensembles. The idea is that screening against several structures might increase the chances of finding the right receptor conformation that accommodate ligand sampling. In a recent paper, Ivetac et al. (2014) described a VS approach using ensembles of experimental and theoretical RT structures to identify novel NNRTIs. A screening library of 2,864 compounds from the National Cancer Institute (NCI) was collected combining compounds from the NCI Diversity Set II (NCIDS-2), a general chemical diversity subset and molecules similar to a set of six known NNRTIs filtered from the NCI repository. The relatively small library was chosen due to the computational demands of the subsequent steps of docking and multistructure docking. An ensemble of diverse RT crystal structures complexed with different NNRTIs was selected to guarantee variation in the conformation of the NNRTI binding site. The screening library was docked, using Glide, into each of the 10 RT structures [PDB codes: 1VRT, 1RT1 (Hopkins et al. 1996), 1HNV (Ding et al. 1995), 1FK9, 1RTH (Ren et al. 1995), 1VRU (Ren et al. 1995), 1EP4, 1BQM (Hsiou et al. 1998), 1KLM (Esnouf et al. 1997) and 2ZD1] and a score was calculated according to the average binding energy of each compound across the ensemble. This score was then used to rank the screening library, favouring molecules that could bind to diverse conformations of the pocket, as opposed to only binding favourably to a particular conformation. MD simulations of 30 ns were performed using the 1VRT structure in complex with four different NNRTIs (each solvated system had approximately 160,000 atoms). Snapshots of the simulations with similar conformations were clustered to ensure representative structures, yielding 30 clusters for each system. From the previous step, only 150 compounds were kept to take part in this secondary screening against all the 120 theoretical conformations. The compounds were ranked and re-scored by taking the mean rank of each compound throughout all simulation systems. Finally, 16 compounds were experimentally tested for inhibition of HIV infection and two of them showed potential inhibition of RT polymerase activity (with potency similar to the positive control NVP). Although successful, this is a very expensive and time-consuming approach. The use of the accurate scoring function is of great importance since a broad range of ligands will be able to fit in some of these more relaxed receptor conformations (Alonso et al. 2006).

This last study is an exceptional example of combining docking methods to MD simulations in a successful way. Given that the use of structures from MD simulations has been successfully employed in docking methods, it has also been investigated the benefits of using multiple of such structures, obtained from a crystallographic one. Nichols et al. (2011)presented an analysis of the usage of structures from MD simulations, with respect to the experimentally determined ones, to improve the predictive power in VS. Two proteins structures were selected, being the HIV-1 RT one of them. Their work consisted in the simulation of two bound systems (PDB code: 1VRT bound with α-APA and UC-781, both NNRTIs) as well as two unbound systems, one with the NNIBP in its open state (PDB code: 1VRT with the NNRTI extracted) and the other with the NNIBP in its closed state (PDB code: 1DLO). All simulations were performed using the GROMACS software along with the GROMOS 53A6 force field (Oostenbrink et al. 2004) and four independent 30 ns trajectories were generated and MD snapshots were extracted. A screening library, consisted of 20 diverse known RTIs combined with a set of 1,323 decoys (compounds from the NCIDS-2) as RT ligands, was prepared. The screening library was docked in each structure, 2,500 MD snapshots for each of the four systems as well as 15 RT X-ray structures (10 diverse NNRTI-bound and 5 NNRTI-free states) using Glide. The predictive power of VS was tested by using the receiver operating characteristic curves (Triballeau et al. 2005), a classification model to establish the probability of ranking active compounds over inactive ones (decoys). This analysis was performed for all different receptor conformations and the results were compared with the ones obtained when employing the same VS approach to the crystallographic structures. In all systems, the maximum MD area under the curve (AUC) value (bound AUC = 0.96 and unbound AUC = 0.77) surpasses the maximum X-ray AUC value (bound AUC = 0.93 and unbound AUC = 0.49). By contrast, when considering mean AUC values, on average bound MD snapshots (<AUC> = 0.76) were less predictive than bound X-ray structures (<AUC> = 0.81), indicating that some MD originated structures had inferior predictive power. However, mean values from unbound systems are equivalent (<AUC> = 0.44 for both). Overall, the advantage of using MD snapshots in VS may depend on the enrichment that a reduced number of MD generated structures provide, rather than the whole configurational ensemble. However, a more accurate method to select the best MD created structure is needed to identify the best conformations and to reduce, time-wise, MD sampling to a more efficient simulation time scale.

In later stages of lead refinement, other calculations might be needed to estimate the relative or absolute free energy of the final complexes. Some of these methods are discussed next.

## Free energy calculations

**Fig. 5:**
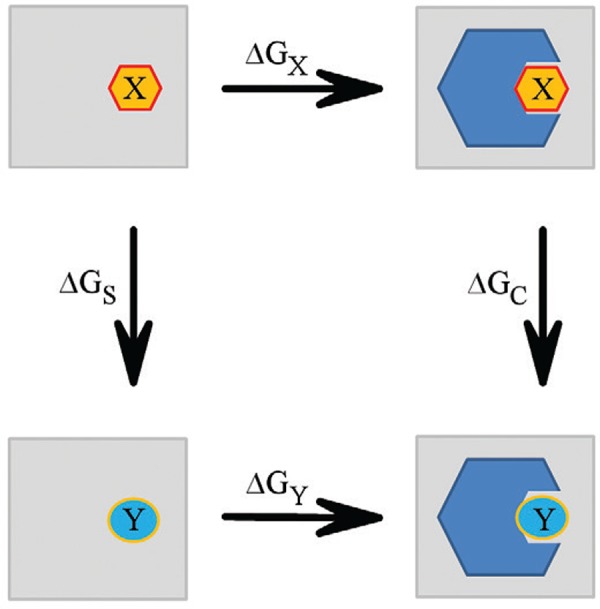
thermodynamic cycle for relative free energies of binding. The receptor is in blue and X and Y are two ligands.

Free energy calculations methodologies are currently employed in several research areas including solvation thermodynamics, molecular recognition and protein folding (Hansen & van Gunsteren 2014). Reviews and applications of free energy calculations in drug design have been described by different authors (Deng & Roux 2009, Michel et al. 2010, Hansen & van Gunsteren 2014). The most rigorous methods to compute relative free energy are free energy perturbation (FEP) and thermodynamic integration (TI) (Pohorille et al. 2010). Using computer aided statistical mechanics, this methods calculate binding free energies of small molecules to a protein through MD or Monte Carlo (MC) simulations (Deng & Roux 2009). For receptor-ligand affinities, perturbations are made to transform one ligand into another using a thermodynamic cycle (Fig. 5). These transformations comprise a coupling parameter that smoothly mutates one molecule to the other. The difference in free energies of binding, from the initial ligand to the final one is calculated by ∆∆G_B_ = ∆G_X_ - ∆G_Y_ = ∆G_S_ - ∆G_C_. To calculate the free energy differences, two transformation systems need to be prepared: one for the unbound ligands in solution (∆G_S_) and the other complexed to the receptor (∆G_C_). These methods can be used to determine the relative free energy, as the free energy is a state function that can be calculated by any reversible path between the initial and final states. Despite their accuracy, these methods are computationally expensive and with slow convergence.

**Fig. 6A:**
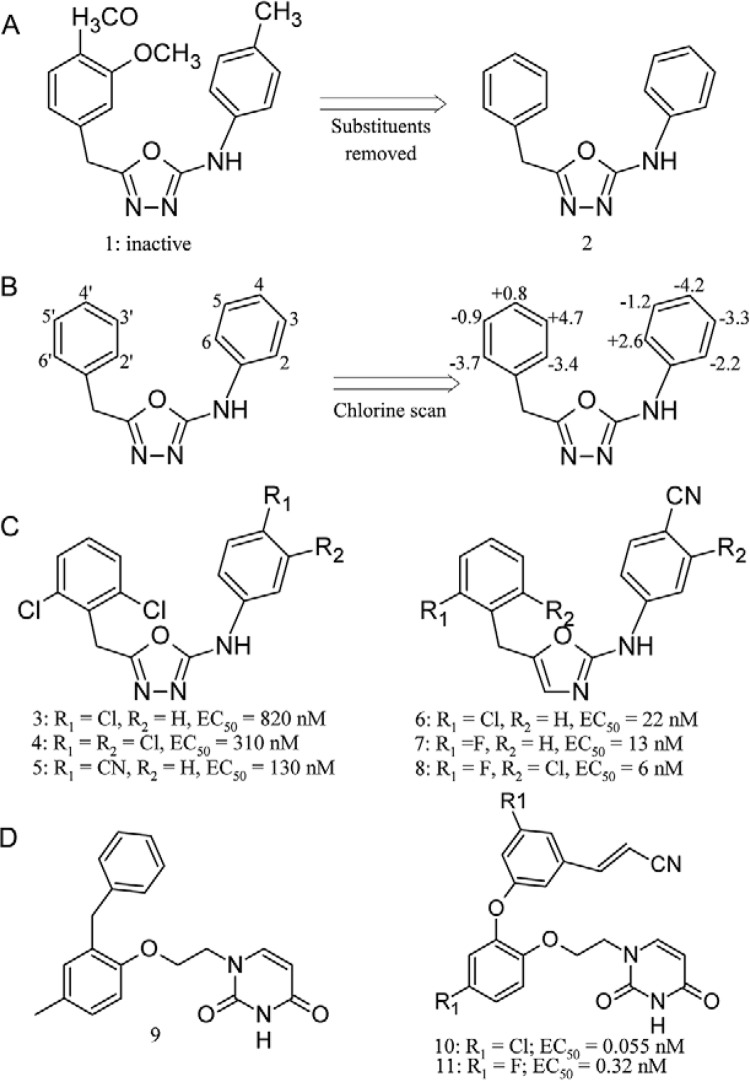
compound 1 and compound 2; B: chlorine scan results performed in compound 2; C, D: resulting compounds from free energy perturbation guided optimisation; EC50: half maximal effective concentration.

Zeevaart et al. (2008), following the early success of searching and optimising of a top scoring compound (1) from a screening library [described Barreiro et al. (2007a)], reported a series of FEP guided simulations with analogues of the modified compound (2), with potencies in the 10-20 nM range. To predict relative free energies of binding, the calculations were carried out in the context of FEP/MC statistical mechanics simulations. These calculations were performed using the thermodynamic cycle theory, to interconvert two ligands unbound in water and bound to the protein. The systems were calculated using dual-topology sampling with 14 windows or simple topology with 11 windows. In FEP calculations, a window refers to a simulation at one point along the mutation coordinate λ, which interconverts two ligands as λ goes from 0-1; the free energy changes are computed for each window, corresponding to a forward and backward increment (the space between windows ∆λ) (Lu et al. 2004). When dual-topology is chosen, the system is prepared in a way that the two complete versions (initial state and final state) of the changing group coexist at every λ (Pearlman 1994). First, a so-called chlorine scan was performed, in which FEP calculations were used to transform each hydrogen individually in the phenyl rings into chlorine, resulting in 10 structures to be converted into compound 2. These FEP results indicated the most promising places for chlorine atoms were at positions 3, 4, 2' and 6' (Fig. 6B). Further optimisation guided the substitution at position 4, resulted in compounds with activity (EC_50_) of 820 nM (3), 310 nM (4) and 130 nM (5) (Fig. 6C). Other FEP scans and ring modifications were made producing compounds with activity (EC_50_) of 22 nM (6), 13 nM (7) and 6 nM (8) (Fig. 6C), the last two found in a later study (Leung et al. 2010).

The same FEP guided optimisation approach was used to improve the performance of a compound, discovered by VS using multiple proteins (Nichols et al. 2009), which showed activity against both WT and Y181C HIV-1 strains. The work by Bollini et al. (2011) started with compound 9, that presented anti-HIV activity EC_50_ of 5 μM and with the aid of FEP/MC outcomes, it was possible to yield a very potent compound (10), EC_50_ values of 55 pM, 42 nM and 220 nM against the WT, the Y181C and K103N/Y181C strains, respectively (Fig. 6D). Further optimisation of compound 10 produced compounds with EC_50_ values of 0.4 nM for the WT and 10 nM for the K103N/Y181C strain (Lee et al. 2013).

Currently developed approaches such as the linear interaction energy method (Aqvist & Marelius 2001) and the so-called MM-Poisson-Boltzmann (PB)/GBSA (MM-PB/GBSA) method (Kollman et al. 2000), both MD-based, provide relatively good free energy predictions at a reasonable cost. Liu et al. (2014) applied the MM-GBSA method to investigate the binding affinities of NVP and two novel NVP analogues when in complex to WT RT (PDB code: 1VRT) and its mutants K103N [PDB code: 1FKP (Ren et al. 2000b)] and Y181C (PDB code: 1JLB). Based on He et al. (2005), whose study showed that NVP binds to the RT through several weak hydrogen bonds, the NVP analogues were constructed in a way that potential strong hydrogen bonds could binding to aas H235 and Y318 in the NNIBP. Alterations were also made to avoid repulsion found in NVP between a carbon atom and the side chain sulfur atom of the mutated C181. Docking was performed to predict the binding mode of the new compounds in the selected structures using AutoDock 4.2 and followed by 20 ns MD simulations for each system with AMBER 11, where snapshots were extracted to obtain ensemble-average binding free energies with the MM-GBSA method. The relative binding free energies of the two NVP analogues (-13.20 and -12.29 kcal/mol) were less favourable than that of NVP (-14.75 kcal/mol) when bound to the WT RT. However, the analogues affinity were more promising to the mutations K103N (-15.57 and -14.76 kcal/mol) and Y181C (-15.50 and -16.32 kcal/mol) than to the NVP affinity when bound to the mutant structures (-12.14 and -10.39 kcal/mol, K103N and Y181C, respectively) and WT. This study showed that these calculations might be helpful to filter and overall assist in the design of new RT drug candidates. Although less computational demanding than FEP or TI, MM-PB/GBSA may not be accurate for the prediction of the entropic component of the free energy and errors might be produced in flexible systems since the internal energy of ligand and receptor upon complex formation are ignored (Alonso et al. 2006).

## QSAR and pharmacophore studies

QSAR is an effort to associate structural or property descriptors of molecules with biological activities quantitatively (Vaidya et al. 2014). The structure-activity relationships are described in terms of physicochemical parameters such as constitutional, fragment constant, thermodynamic, conformational, hydrophobicity, topology, electronic properties, steric effects, hydrogen bond donors, hydrogen bond acceptors, among others (Kubinyi & Sadowski 1999). These descriptors can be determined empirically or by computational methods. Computational QSAR studies are often used to filter virtually large compounds libraries, to eliminate the molecules with predicted toxic or poor pharmacokinetic properties early on and to narrow the libraries to drug-like or lead-like compounds (Dudek et al. 2006). Some QSAR studies applied to RT have been reported (Gaudio & Montanari 2002, Gayen et al. 2004, Guimarães et al. 2014, Dong & Ren 2015, Nazar et al. 2015).

Recently, Tarasova et al. (2015) published a study addressing the use of data from publicly and commercially available databases to produce accurate and predictive QSAR models using RT as the case study. Two databases, Thomson Reuters' Integrity and ChEMBL (Bento et al. 2014), were chosen to collect all RTIs assayed against both WT and mutants RT. Several methods for the creation of modelling sets from the chosen databases were proposed and their accuracy investigated. The program GUSAR was used to build the QSAR models. From the Integrity database, when the compilation of modelling sets were according to their assay data (i.e., associated with just one material and method for testing), it yielded high-performance QSAR models for all RT forms. While ChEMBL database, compounds derived from individual scientific publications provided more consistent and higher quality QSAR models than the other methods employed in the same database. Although, some of the methods worked within the databases it did not work across them in a mix-and-match QSAR model approach. The lack of unified and standardised descriptors between Integrity and ChEMBL revealed to be a problem to data aggregation.

Li et al. (2008) published a paper that combined 3D-QSAR with MD simulations. The 3D-QSAR methodology consists of obtaining compound descriptors from an experimentally determined ligand and aligning conformers of the chosen dataset in space (Dudek et al. 2006). A series of diaryltriazine analogues, a category of NNRTIs, were extracted from the literature and separated in two data sets (8A-G and 9A-R) based on their structure and activity (IC_50_). The steric and electrostatic interactions were calculated by the comparative molecular field analysis (MFA) method and compounds with low, moderate and high activity were selected. Five physicochemical properties related to steric, electrostatic, hydrophobic, hydrogen bond donor and hydrogen bond acceptor parameters were evaluated by the comparative molecular similarity analysis method. The two most active molecules, one from each data set, 8E and 9H (pIC_50_ of 1.21 and 2.04, respectively), were further analysed by 2.0 ns MD simulations. The simulations showed more hydrophobic contacts and hydrogen bonds for 9H, which might make it more active and stable than 8E. However, superposition of these two compounds showed similar binding modes with the RT, indicating that a conventional 3D-QSAR model of these two types of RTI could be constructed.

Another method frequently used in drug discovery is pharmacophore modelling. In it, the steric and electronic features a query molecule possesses, essential for receptor-ligand interaction, is analysed (Zheng et al. 2013). The resulting model can be determined either based on ligand information, by superposing a set of active molecules and selecting essential common features for their bioactivity or based on structural information. The method helps in the search for possible interaction points identified between receptor and ligands (Yang 2010). Some pharmacophore studies have been reported for RTIs (Keller et al. 2003, Balaji et al. 2004, Distinto et al. 2012).

Vadivelan et al. (2011) developed a work where potential anti-HIV lead compounds could be generated by analogue based design studies using 3D-QSAR and pharmacophore models. A training set of 36 molecules was used to develop the best model. The MFA model was generated based on the feature query of the biologically active conformation of the most active compound (pIC_50_ of 8.57). The best pharmacophore model was composed of three characteristics: one hydrogen bond acceptor, one hydrophobic aliphatic and one aromatic ring. Both models were validated and their predictive ability evaluated by knowledge-based screening. A total of 10,000 molecules were generated based on the knowledge of the binding interaction of ligands to the RT and also the common features necessary for the molecule biological activity. Cross validation was made with both models and the results suggested that these techniques yield almost the same results. However, the screening produced some false-positives and a few false-negatives. Therefore, docking studies were performed on a RT structure [PDB code: 2VG5 (Spallarossa et al. 2008)] using Glide in an attempt to produce reliable true positives and negatives. The combined approach developed in their study showed a possible way to assess critically the identification and optimisation of lead compounds through better understanding of protein and ligand features.

## ADMET studies

ADMET studies are commonly applied in drug discovery to optimise leads compounds into drug candidates (Selick et al. 2002). Experimental ADMET investigations allow classifying based on characteristics such as the ability to cross physiological barriers, group reactivity, metabolism and so on (Oprea et al. 2001, Selick et al. 2002, Kubinyi 2003). *In silico* computations can be carried out to analyse the drug-likeness of a compound prior to its synthesis (Beresford et al. 2004). A series of filtering rules are defined to compute what are called descriptors that classify the compounds and to predict their ADMET properties (Lagorce et al. 2008). While these descriptors are not accurate enough to replace in vivo or in vitro methods, they can help point out physicochemical properties and lead to the optimisation of them (Gleeson et al. 2011).

An early work from Sengupta et al. (2007)analysed 15 DLV analogues for their potential to be used as drug candidates. Their approach consisted of docking the compounds to determine an initial binding mode of the ligand with the receptor. Then, free energy calculations with MM-GBSA were performed. Finally, ADME properties were estimated by Qikprop (Duffy & Jorgensen 2000). The program predicted 44 properties consisting of principal descriptors and physiochemical properties such as log P (Octanol/Water), log P Madin-Darby canine kidney (MDCK) (predicted apparent MDCK cell permeability) and log K_p_ (skin permeability). Violations of the Lipinski's rule of five were also considered. From this analysis, 15 out of the 16 compounds showed acceptable values for all the properties analysed. Based on the overall examination, three analogues showed potential as a leads to be used for drug development. These three compounds exhibited efficient binding in the active site, showing ideal pIC_50_ (~7.0) values and passed the rule of five. This work demonstrated the use of ADME properties as a tool to aggregate value to suitable candidates for drug development.

Pirhadi and Ghasemi (2012) used a combination of pharmacophore model for NNRTIs, docking and ADME studies in the search for novel compounds. Firstly, a set of 219 compounds comprising diverse structures was obtained. Based on these compounds, quantitative pharmacophore models were developed to identify critical features among NNRTIs. The best pharmacophore model took into account four descriptors, including two hydrogen bond acceptors, one hydrophobic and one aromatic feature, in agreement with previously reported pharmacophore models. The model was used as a 3D VS query for recovering novel and potent candidates from ZINC (Irwin & Shoichet 2005), resulting in 8,631 hits from this first screening. Next, this set was filtered based on pharmacokinetic properties (Lipinski's rule of 5) and the 6,229 molecules that remained were then docked into the NNRTI binding pocket of the RT structure [PDB code: 3DLG (Ren et al. 2008)]. Seven compounds were retrieved and submitted for ADME prediction studies. Nearly all the structures presented acceptable values for the ADME properties analysed, such as log K_p_, apparent Caco-2 and MDCK permeability, log BB (predicted brain/blood partition coefficient), aqueous solubility (log S), maximum of transdermal transport rate (J_m_), human oral absorption in the gastrointestinal tract, log K_hsa_ for serum protein binding and log P. No experimental results were reported in the paper. However, their approach seemed to favour high potency compounds since three of the compounds are available in the ChEMBL database with varied but high reported potency, yet none of the potency reported was against the RT.

## Concluding remarks

In the last two decades, substantial advances have been made in development of novel antiretroviral drugs. The newest FDA approved drugs, ETR (2008, NNRTI), RPV (2011, NNRTI), dolutegravir (2013, integrase inhibitor) and elvitegravir (2014, integrase inhibitor) indicate recent research efforts to the current antiretroviral drug classes. However, the emergence of drug-resistance strains call for not only new classes of anti-HIV drugs with lower toxicity and favourable resistance profile, but also innovative drug discovery strategies for antiretroviral treatment. For instance, a few compounds targeting the existing classes are in advanced stages of development: TFV alafenamide fumarate is a pro-drug of TFV, currently in Phase 3 of clinical trials, which seems to have less renal and bone toxicity than its precursor (Sax et al. 2014); the NNRTI doravirine (MK-1439), currently in Phase 2, exhibits activity against resistant viral strains (Gatell et al. 2014) and an integrase inhibitor currently in Phase 2, GSK1265744 (an experimental analogue of dolutegravir), is being established in a long-acting preparation (Spreen et al. 2013). In addition, novel explored alternatives to prevention and progression, such as microbicides (Buckheit et al. 2010), antiretroviral prophylaxis (Karim & Karim 2012), CD4-mimetic compounds (Gardner et al. 2015,Richard et al. 2015) and broadly neutralising HIV-specific antibodies (Diskin et al. 2011, Moir et al. 2011, McCoy & Weiss 2013), show potential for reducing HIV-1 transmission rates. Investigational drugs such as the CD4 attachment inhibitor BMS-663068 completed Phase 1 testing (Nettles et al. 2012) and cenicriviroc, a novel CCR5/CCR2 antagonist currently in Phase 3 (Klibanov et al. 2010), that suggests both antiretroviral activity and potential for an antiinflammatory effect.

Although HIV-1 RT is an extremely validated target, which has been widely studied, the discovery of novel allosteric sites and alternative mechanisms to this enzyme provide insights to develop new therapeutic classes of inhibitors (Kang et al. 2014). Consequently, inhibitors with distinct mechanisms have been exploited and can be found in the literature, comprising, RT-directed mutagenic inducers (Smith et al. 2005), nucleotide-competing RTIs (Maga et al. 2010), RT-associated RNase H function inhibitors (Yu et al. 2008), primer/template-competing RTIs (Wang et al. 2004), dual inhibitors of the RT associated polymerase and RNase H activities (Esposito et al. 2011) and NNRTIs with not-conventionally-binding modes or alternative mechanisms (Pata et al. 2004,Cullen et al. 2009, Zhan et al. 2010, Das et al. 2011). However, the majority of these inhibitors have not been explored by means of computational methods.

The application of computational methods is of great importance to drug discovery nowadays and it is principally beneficial to investigate drug resistance development. Methods such as molecular docking, MD, free energy calculations, QSAR, pharmacophore modelling and ADMET are broadly applied in anti-HIV drug development. The focus of this review was the HIV-1 RT; however, the approaches discussed are also in use when targeting other HIV proteins. The extensive research targeting the RT throughout the years has benefited from the employment of computational methods, extracting information from the currently available compounds and crystallographic structures to generate many successful stories in inhibitor discovery and optimisation. The computational methods employed provide beneficial results that can expand and guide the drug discovery process in all stages.
